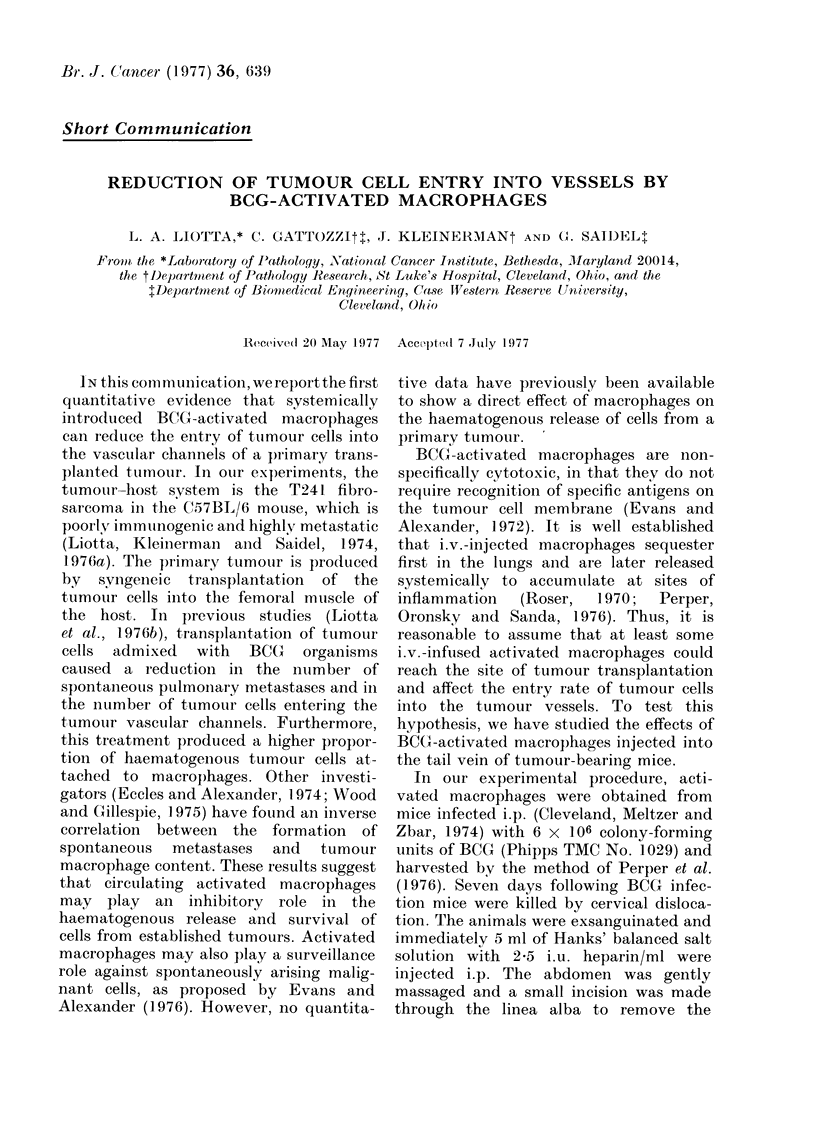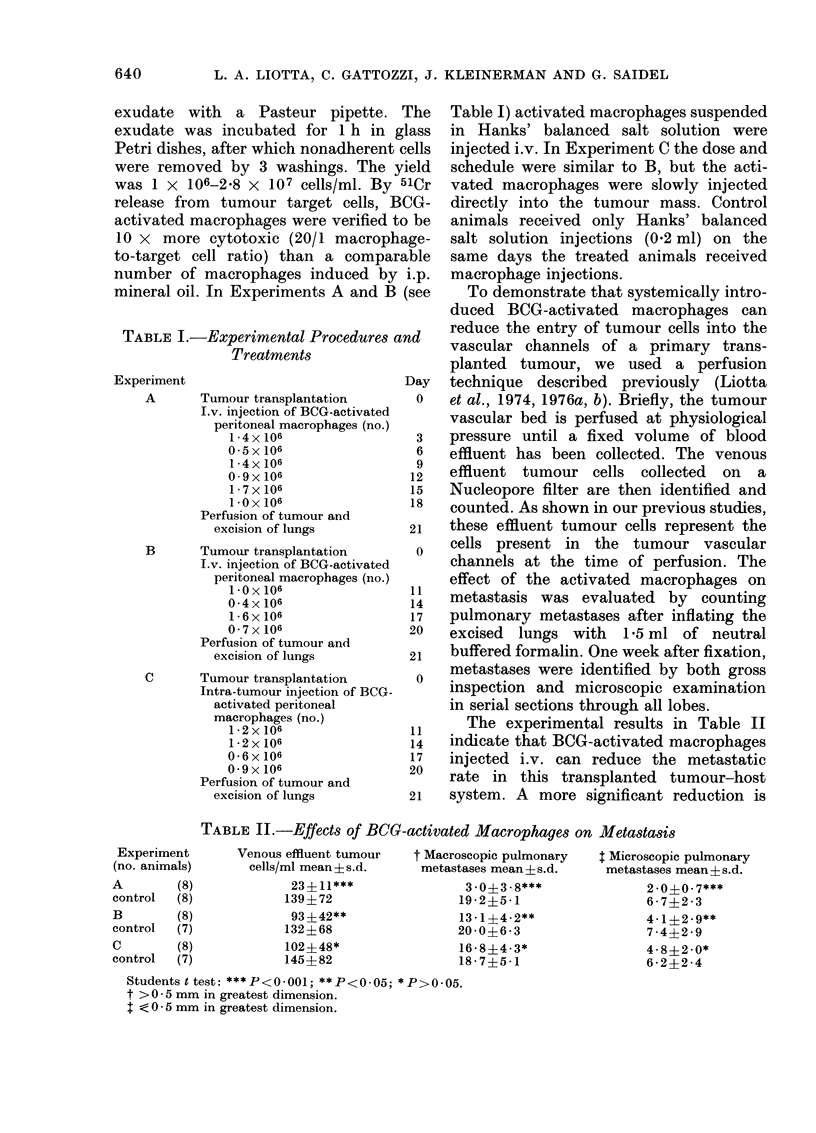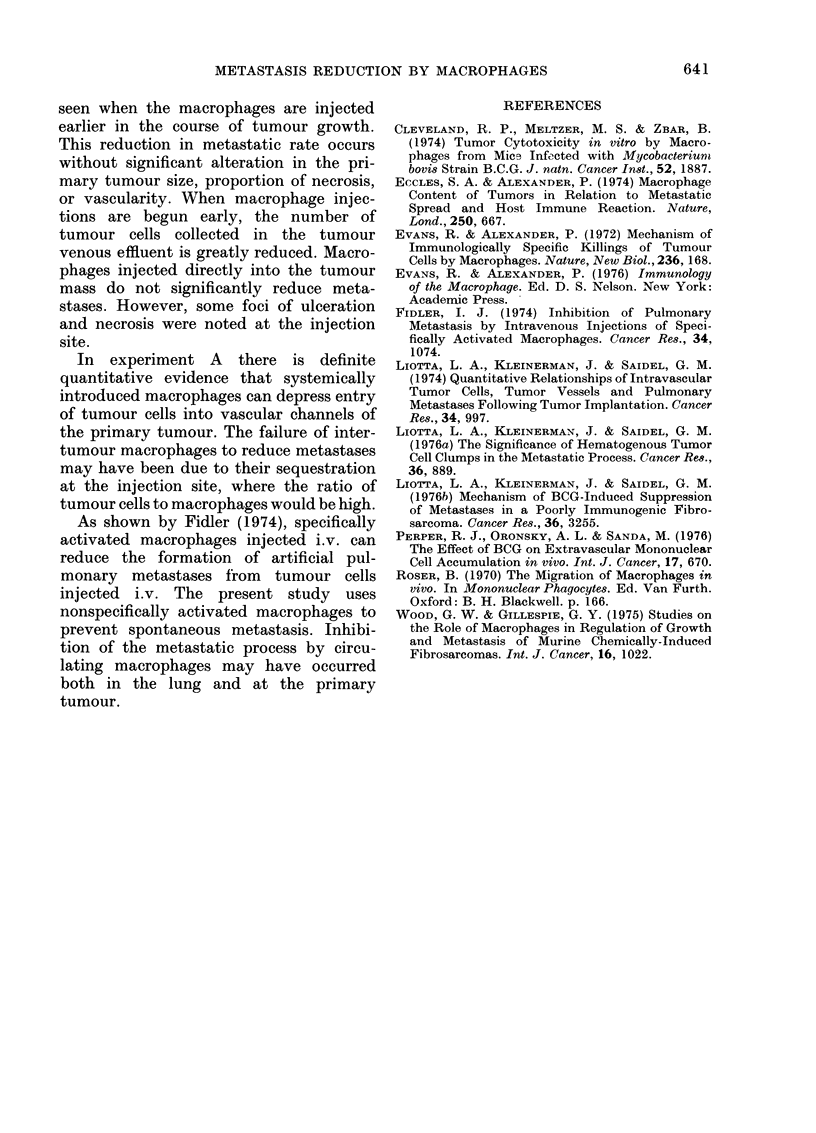# Reduction of tumour cell entry into vessels by BCG-activated macrophages.

**DOI:** 10.1038/bjc.1977.242

**Published:** 1977-11

**Authors:** L. A. Liotta, C. Gattozzi, J. Kleinerman, G. Saidel


*Br. J. Cancer* (1977) **36**, 639

***Short Communication***

### REDUCTION OF TUMOUR CELL ENTRY INTO VESSELS BY BCG-ACTIVATED MACROPHAGES

L. A. LIOTTA,* C. GATTOZZI†‡, J. KLEINERMAN† AND G. SAIDEL‡

*From the *Laboratory of Pathology, National Cancer Institute, Bethesda, Maryland* 20014, *the †Department of Pathology Research, St Luke's Hospital, Cleveland, Ohio, and the ‡Department of Biomedical Engineering, Case Western Reserve University, Cleveland, Ohio*

Received 20 May 1977 Accepted 7 July 1977

In this communication, we report the first quantitative evidence that systemically introduced BCG-activated macrophages can reduce the entry of tumour cells into the vascular channels of a primary transplanted tumour. In our experiments, the tumour–host system is the T241 fibrosarcoma in the C57BL/6 mouse, which is poorly immunogenic and highly metastatic (Liotta, Kleinerman and Saidel, 1974, 1976*a*). The primary tumour is produced by syngeneic transplantation of the tumour cells into the femoral muscle of the host. In previous studies (Liotta *et al.*, 1976*b*), transplantation of tumour cells admixed with BCG organisms caused a reduction in the number of spontaneous pulmonary metastases and in the number of tumour cells entering the tumour vascular channels. Furthermore, this treatment produced a higher proportion of haematogenous tumour cells attached to macrophages. Other investigators (Eccles and Alexander, 1974; Wood and Gillespie, 1975) have found an inverse correlation between the formation of spontaneous metastases and tumour macrophage content. These results suggest that circulating activated macrophages may play an inhibitory role in the haematogenous release and survival of cells from established tumours. Activated macrophages may also play a surveillance role against spontaneously arising malignant cells, as proposed by Evans and Alexander (1976). However, no quantitative data have previously been available to show a direct effect of macrophages on the haematogenous release of cells from a primary tumour.

BCG-activated macrophages are nonspecifically cytotoxic, in that they do not require recognition of specific antigens on the tumour cell membrane (Evans and Alexander, 1972). It is well established that i.v.-injected macrophages sequester first in the lungs and are later released systemically to accumulate at sites of inflammation (Roser, 1970; Perper, Oronsky and Sanda, 1976). Thus, it is reasonable to assume that at least some i.v.-infused activated macrophages could reach the site of tumour transplantation and affect the entry rate of tumour cells into the tumour vessels. To test this hypothesis, we have studied the effects of BCG-activated macrophages injected into the tail vein of tumour-bearing mice.

In our experimental procedure, activated macrophages were obtained from mice infected i.p. (Cleveland, Meltzer and Zbar, 1974) with $6 \times 10^6$ colony-forming units of BCG (Phipps TMC No. 1029) and harvested by the method of Perper *et al.* (1976). Seven days following BCG infection mice were killed by cervical dislocation. The animals were exsanguinated and immediately 5 ml of Hanks' balanced salt solution with 2·5 i.u. heparin/ml were injected i.p. The abdomen was gently massaged and a small incision was made through the linea alba to remove the exudate with a Pasteur pipette. The exudate was incubated for 1 h in glass Petri dishes, after which nonadherent cells were removed by 3 washings. The yield was $1 \times 10^6$–$2{\cdot}8 \times 10^7$ cells/ml. By $^{51}Cr$ release from tumour target cells, BCG-activated macrophages were verified to be 10 × more cytotoxic (20/1 macrophage-to-target cell ratio) than a comparable number of macrophages induced by i.p. mineral oil. In Experiments A and B (see Table I) activated macrophages suspended in Hanks' balanced salt solution were injected i.v. In Experiment C the dose and schedule were similar to B, but the activated macrophages were slowly injected directly into the tumour mass. Control animals received only Hanks' balanced salt solution injections (0·2 ml) on the same days the treated animals received macrophage injections.

TABLE I.—*Experimental Procedures and Treatments*

| Experiment | | Day |
|---|---|---|
| A | Tumour transplantation | 0 |
| | I.v. injection of BCG-activated peritoneal macrophages (no.) | |
| | $1{\cdot}4\times10^6$ | 3 |
| | $0{\cdot}5\times10^6$ | 6 |
| | $1{\cdot}4\times10^6$ | 9 |
| | $0{\cdot}9\times10^6$ | 12 |
| | $1{\cdot}7\times10^6$ | 15 |
| | $1{\cdot}0\times10^6$ | 18 |
| | Perfusion of tumour and excision of lungs | 21 |
| B | Tumour transplantation | 0 |
| | I.v. injection of BCG-activated peritoneal macrophages (no.) | |
| | $1{\cdot}0\times10^6$ | 11 |
| | $0{\cdot}4\times10^6$ | 14 |
| | $1{\cdot}6\times10^6$ | 17 |
| | $0{\cdot}7\times10^6$ | 20 |
| | Perfusion of tumour and excision of lungs | 21 |
| C | Tumour transplantation | 0 |
| | Intra-tumour injection of BCG-activated peritoneal macrophages (no.) | |
| | $1{\cdot}2\times10^6$ | 11 |
| | $1{\cdot}2\times10^6$ | 14 |
| | $0{\cdot}6\times10^6$ | 17 |
| | $0{\cdot}9\times10^6$ | 20 |
| | Perfusion of tumour and excision of lungs | 21 |

To demonstrate that systemically introduced BCG-activated macrophages can reduce the entry of tumour cells into the vascular channels of a primary transplanted tumour, we used a perfusion technique described previously (Liotta *et al.*, 1974, 1976*a*, *b*). Briefly, the tumour vascular bed is perfused at physiological pressure until a fixed volume of blood effluent has been collected. The venous effluent tumour cells collected on a Nucleopore filter are then identified and counted. As shown in our previous studies, these effluent tumour cells represent the cells present in the tumour vascular channels at the time of perfusion. The effect of the activated macrophages on metastasis was evaluated by counting pulmonary metastases after inflating the excised lungs with 1·5 ml of neutral buffered formalin. One week after fixation, metastases were identified by both gross inspection and microscopic examination in serial sections through all lobes.

The experimental results in Table II indicate that BCG-activated macrophages injected i.v. can reduce the metastatic rate in this transplanted tumour–host system. A more significant reduction is

TABLE II.—*Effects of BCG-activated Macrophages on Metastasis*

| Experiment (no. animals) | | Venous effluent tumour cells/ml mean ± s.d. | † Macroscopic pulmonary metastases mean ± s.d. | ‡ Microscopic pulmonary metastases mean ± s.d. |
|---|---|---|---|---|
| A | (8) | 23 ± 11*** | 3·0 ± 3·8*** | 2·0 ± 0·7*** |
| control | (8) | 139 ± 72 | 19·2 ± 5·1 | 6·7 ± 2·3 |
| B | (8) | 93 ± 42** | 13·1 ± 4·2** | 4·1 ± 2·9** |
| control | (7) | 132 ± 68 | 20·0 ± 6·3 | 7·4 ± 2·9 |
| C | (8) | 102 ± 48* | 16·8 ± 4·3* | 4·8 ± 2·0* |
| control | (7) | 145 ± 82 | 18·7 ± 5·1 | 6·2 ± 2·4 |

Students *t* test: *** $P<0{\cdot}001$; ** $P<0{\cdot}05$; * $P>0{\cdot}05$.

† $>0{\cdot}5$ mm in greatest dimension.

‡ $\leqslant 0{\cdot}5$ mm in greatest dimension.

seen when the macrophages are injected earlier in the course of tumour growth. This reduction in metastatic rate occurs without significant alteration in the primary tumour size, proportion of necrosis, or vascularity. When macrophage injections are begun early, the number of tumour cells collected in the tumour venous effluent is greatly reduced. Macrophages injected directly into the tumour mass do not significantly reduce metastases. However, some foci of ulceration and necrosis were noted at the injection site.

In experiment A there is definite quantitative evidence that systemically introduced macrophages can depress entry of tumour cells into vascular channels of the primary tumour. The failure of intertumour macrophages to reduce metastases may have been due to their sequestration at the injection site, where the ratio of tumour cells to macrophages would be high.

As shown by Fidler (1974), specifically activated macrophages injected i.v. can reduce the formation of artificial pulmonary metastases from tumour cells injected i.v. The present study uses nonspecifically activated macrophages to prevent spontaneous metastasis. Inhibition of the metastatic process by circulating macrophages may have occurred both in the lung and at the primary tumour.

### REFERENCES

Cleveland, R. P., Meltzer, M. S. & Zbar, B. (1974) Tumor Cytotoxicity *in vitro* by Macrophages from Mice Infected with *Mycobacterium bovis* Strain B.C.G. *J. natn. Cancer Inst.*, **52**, 1887.

Eccles, S. A. & Alexander, P. (1974) Macrophage Content of Tumors in Relation to Metastatic Spread and Host Immune Reaction. *Nature, Lond.*, **250**, 667.

Evans, R. & Alexander, P. (1972) Mechanism of Immunologically Specific Killings of Tumour Cells by Macrophages. *Nature, New Biol.*, **236**, 168.

Evans, R. & Alexander, P. (1976) *Immunology of the Macrophage*. Ed. D. S. Nelson. New York: Academic Press.

Fidler, I. J. (1974) Inhibition of Pulmonary Metastasis by Intravenous Injections of Specifically Activated Macrophages. *Cancer Res.*, **34**, 1074.

Liotta, L. A., Kleinerman, J. & Saidel, G. M. (1974) Quantitative Relationships of Intravascular Tumor Cells, Tumor Vessels and Pulmonary Metastases Following Tumor Implantation. *Cancer Res.*, **34**, 997.

Liotta, L. A., Kleinerman, J. & Saidel, G. M. (1976*a*) The Significance of Hematogenous Tumor Cell Clumps in the Metastatic Process. *Cancer Res.*, **36**, 889.

Liotta, L. A., Kleinerman, J. & Saidel, G. M. (1976*b*) Mechanism of BCG-Induced Suppression of Metastases in a Poorly Immunogenic Fibrosarcoma. *Cancer Res.*, **36**, 3255.

Perper, R. J., Oronsky, A. L. & Sanda, M. (1976) The Effect of BCG on Extravascular Mononuclear Cell Accumulation *in vivo*. *Int. J. Cancer*, **17**, 670.

Roser, B. (1970) The Migration of Macrophages *in vivo*. In *Mononuclear Phagocytes*. Ed. Van Furth. Oxford: B. H. Blackwell. p. 166.

Wood, G. W. & Gillespie, G. Y. (1975) Studies on the Role of Macrophages in Regulation of Growth and Metastasis of Murine Chemically-Induced Fibrosarcomas. *Int. J. Cancer*, **16**, 1022.